# Genetically predicted the causal relationship between gut microbiota and the risk of polymyositis/dermatomyositis: a Mendelian randomization analysis

**DOI:** 10.3389/fmicb.2024.1409497

**Published:** 2024-08-21

**Authors:** Yanna Niu, Yaochen Zhang, Keyi Fan, Jialin Hou, Liu Liu, Heyi Zhang, Xinlei Geng, Xiyue Ma, Shilei Lin, Meilin Guo, Xiaofeng Li, Shengxiao Zhang

**Affiliations:** ^1^Department of Rheumatology, The Second Hospital of Shanxi Medical University, Taiyuan, Shanxi, China; ^2^Shanxi Provincial Key Laboratory of Rheumatism Immune Microecology, Taiyuan, Shanxi, China; ^3^Key Laboratory of Cellular Physiology at Shanxi Medical University, Ministry of Education, Taiyuan, Shanxi, China; ^4^SXMU-Tsinghua Collaborative Innovation Center for Frontier Medicine, Shanxi medical university, Taiyuan, Shanxi, China

**Keywords:** causal relationship, dermatomyositis, gut microbiota, Mendelian randomization, polymyositis

## Abstract

**Introduction:**

Observational studies suggest associations between gut microbiota and polymyositis (PM) and dermatomyositis (DM), but causal relationships are unclear. We investigate the causal effects of gut microbiota on PM and DM, providing insights hoping to provide insights for future treatment and prevention.

**Methods:**

Summary statistics of gut microbiota were obtained from a multi-ethnic Genome Wide Association Studies (GWAS) meta-analysis, including 119 taxa from 18,340 Europeans. PM/DM statistics were extracted from GWAS analyses. Mendelian randomization (MR) with IVW, MR-Egger, and weighted median methods was performed. Sensitivity analyses addressed heterogeneity and pleiotropy. Of the 119 bacterial genera studied, six showed causal links.

**Results:**

*Alloprevotella* (OR: 3.075, 95% CI: 1.127–8.386, *p* = 0.028), *Ruminococcaceae* UCG003 (OR: 4.219, 95% CI: 1.227–14.511, *p* = 0.022), *Dialister* (OR: 0.273, 95% CI: 0.077–0.974, *p* = 0.045) were associated with PM. *Anaerotruncus* (OR: 0.314, 95% CI: 0.112–0.882, *p* = 0.028), *Ruminococcaceae* UCG002 (OR: 2.439, 95% CI: 1.173–5.071, *p* = 0.017), *Sutterella* (OR: 3.392, 95% CI: 1.302–8.839, *p* = 0.012) were related to DM. Sensitivity analyses validated these associations

**Discussion:**

We establish causal relationships between *Ruminococcaceae*, *Sutterella*, *Anaerotruncus* with DM, *Alloprevotella*, *Ruminococcaceae* UCG003, and *Dialister* with PM. Common microbiota, like *Ruminococcaceae*, have significant clinical implications. These findings open up greater possibilities for the gut microbiota to contribute to the development of PM/DM and for future monitoring of the gut microbiota in patients with PM/DM.

## Introduction

Polymyositis (PM) and dermatomyositis (DM) are autoimmune muscle diseases characterized by an infiltration of inflammatory cells in the muscle tissue (Ricroch and Hénard-Damave, 2016), and both belong to the category of idiopathic inflammatory myopathies (IIM) (Findlay et al., 2015). The incidence rates for these diseases range from 4.27 and 7.89 per 100,000 person-years, with prevalence rates ranging from 9.54 to 32.74 cases per 100,000 individuals (Bernatsky et al., 2009; Furst et al., 2012; Smoyer-Tomic et al., 2012). PM is more common in women and African Americans, while DM primarily affects children and adults (Amato and Barohn, 2009). Recent studies have implicated the potential role of the gut microbiota in the development of various autoimmune diseases, including PM and DM (Findlay et al., 2015). Understanding the underlying mechanisms is critical for future treatment and prevention of PM/DM (Li et al., 2021).

The gut microflora has always been a more popular topic (De Luca and Shoenfeld, 2019). Compared to other parts of the body, the intestinal flora has the largest number and variety of bacteria (Zhufeng et al., 2022), with about 35,000 species. Human gut microbes are determined by a combination of genetic, epigenetic, and dietary factors. Interestingly, several studies have shown that gut microbiota plays a key role in autoimmune diseases by altering the abundance of microbial metabolites for immunomodulatory functions (Findlay et al., 2015). In particular, the Firmicutes (FC) and Bacteroidetes (BT) phyla play important roles in nutrient metabolism, antimicrobial protection, and immunomodulation (Geuking et al., 2011; Chung et al., 2012; Jandhyala et al., 2015). Previous studies have shown that the human gut microbiota is determined by a combination of genetic, epigenetic, and dietary factors. Recent studies have revealed the potential role of gut microbiota dysbiosis in the pathogenesis of various autoimmune diseases, including PM and DM (Findlay et al., 2015). Moreover, the dysregulation of gut microbiota has been associated with several human diseases, such as inflammatory bowel disease (IBD), metabolic disorders like diabetes and obesity, allergic diseases, and neurodevelopmental disorders (Bisgaard et al., 2011; Karlsson et al., 2013; Ferreira et al., 2014). However, the specific causal relationship between different gut microorganisms and the development of PM/DM remains uncertain and requires further research (Liu et al., 2023).

To address the need for causal inference and identify the primary influencing factors within the gut microbiome, we conducted a Mendelian randomization (MR) analysis. MR analysis was employed as a driving force for further research to meet the need for causal inference and to identify key factors influencing the gut microbiome. By utilizing genetic instruments to minimize confounding factors, MR analysis enables the assessment of causal effects and aims to uncover the potential relationship between gut microbiota and polymyositis (PM) and dermatomyositis (DM). This analysis furthers understanding of the role of gut flora in PM/DM by identifying relevant gut microbes to facilitate early disease screening, implementation of interventions, and personalized healthcare (Bowden and Holmes, 2019).

Previous observational studies have emphasized the link between reduced microbial diversity and DM, but a causal relationship has not been established (Zhufeng et al., 2022). In addition, there is a dearth of research on the key gut microbiota that may impact the development of PM. This study aims to elucidate the specific gut microflora that potentially contribute to the onset of PM/DM and assess their potential as novel targets for treatment. Notably, as muscle biopsy is invasive and required for definitive PM/DM diagnosis (Findlay et al., 2015), the non-invasive, convenient, and rapid fecal testing for gut flora offers promising opportunities. It is anticipated that the use of fecal testing of intestinal flora will enable early identification of patients with PM/DM and provide personalized therapeutic interventions.

## Materials and methods

### Study design

**Figure 1:**
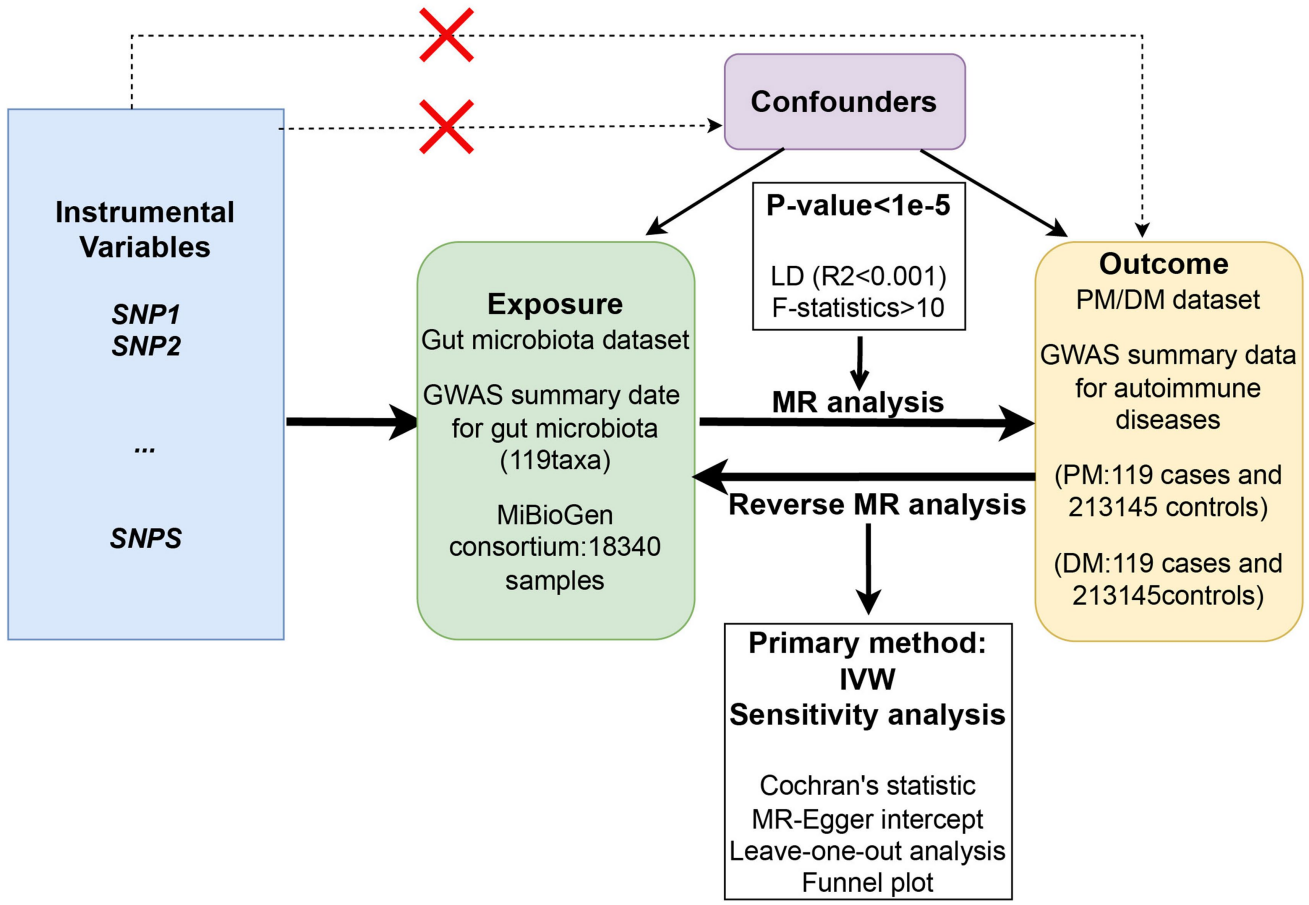
The overall flowchart of the study design of the two-sample Mendelian randomization for the effect of genetically predicted gut microbiome on PM/DM.

This study employs a two-sample Mendelian randomization (MR) analysis, utilizing summary statistics from Genome Wide Association Studies (GWAS), to explore the potential causal effect of gut microbiota on PM and DM. This MR analysis was conducted following the Strengthening the Reporting of Observational Studies in Epidemiology using Mendelian Randomization (STROBE-MR) Statement (Skrivankova et al., 2021a,b). Single nucleotide polymorphisms (SNPs) significantly associated with the gut microbiota were selected as instrumental variables (IVs), and three assumptions were made to ensure accuracy. First, the genetic variants selected as IVs should be significantly associated with gut microbiota. Second, IVs should not be associated with any confounding factors. Third, IVs should affect the risk of PM and DM only through gut microbiota. A series of sensitivity analyses were then conducted for significant associations. The overall flowchart of the study is presented in Figure 1.

### Genetic datasets

Summary statistics of the gut microbiota were obtained from a large-scale multi-ethnic GWAS meta-analysis, involving 18,340 individuals from 24 cohorts, most of which were of European ancestry (Kurilshikov et al., 2021). Genus-level taxa were analyzed, with a total of 131 genera showing an average abundance greater than 1%. However, 12 of these genera were unknown, leaving 119 genus-level taxa included in this study. Covariate adjustments were made for gender, age, technical covariates, and genetic principal components using Spearman correlation analysis.

The outcome datasets were extracted from the large publicly available GWAS analysis. Summary data for DM were obtained from a GWAS study of European ancestry, including 208 cases and 213,145 controls. For polymyositis (PM), the study included 119 cases and 213,145 controls.

### Selection of IVs

Prior to the MR analysis, a rigorous screening process was conducted to ensure the reliability of the SNPs and meet the assumptions of MR analysis. The IVs were selected based on SNPs associated with gut microbiota at a genome-wide significance level (*p* < 1 × 10^−5^). A linkage disequilibrium (LD) aggregation threshold of *r*^2^ < 0.001 was applied to remove correlated and dependent SNPs to ensure independence and validity (Li et al., 2023). To assess potential confounding effects, the selected SNPs were evaluated for associations with other phenotypes using the publicly available PhenoScanner V2^1^ (Kamat et al., 2019). SNPs related to any potential confounders were removed at genome-wide significance. Additionally, only SNPs with consistent effect alleles between the exposure and outcome GWAS datasets were included, while those absent in the outcome GWAS were removed. In addition, to eliminate the bias of weak IVs, we calculated the *F*-statistic for SNPs using the following formula: *F* = *r*^2^(*n* − *k* − 1)/[*k*(1 − *r*^2^)]. the value of the *F*-statistic indicates the strength of the IV, and *F*-statistic >10 is a strong IV (Ni et al., 2021).

### Statistical analysis

#### MR analysis

To assess the causal relationship between gut microflora and outcomes, MR analysis was conducted using three methods: inverse variance weighting (IVW), MR-Egger regression, and weighted median (WME). These methods have been extensively described in previous studies (Didelez and Sheehan, 2007; Burgess et al., 2017). IVW is essentially a meta-analytic approach that translates into a weighted regression of the outcome effects of instrumental variables on exposure effects to obtain an overall estimate of the impact of the gut microbiome on PM/DM risk, where the intercept is restricted to zero (Choi et al., 2019). When there is no horizontal pleiotropy, IVW avoids the effects of confounders and obtains unbiased estimates. Besides, IVW analysis was considered the most reliable method for estimating the causal relationship between exposure and outcome while being sensitive to pleiotropy. MR-Egger regression and WME were further applied as complementary analyses (Burgess et al., 2017). For MR-egger, the traditional Mendelian approach to randomization analysis assumes that all genetic variants satisfy the instrumental variable assumptions (Burgess and Thompson, 2017). However, MR-Egger regression, which has the property of detecting and adjusting for pleiotropy in MR analysis and obtaining causal effect estimates (Bowden et al., 2016), and checking whether the results are driven by directional horizontal pleiotropy (Burgess and Thompson, 2017). Even if under an assumption that is weaker than standard instrumental variable assumptions, the slope coefficient from the Egger regression method provides an estimate of the causal effect that is consistent asymptotically even if all the genetic variants have pleiotropic effects on the outcome (Bowden et al., 2015, 2016). This is the assumption that the pleiotropic effect of a genetic variant (i.e., the direct effect of the genetic variant on the outcome without acting through risk factors) is independent of instrument strength (known as the InSIDE assumption Instrument Strength Is Independent of Direct Effect). At the same time, the MR-Egger regression provides a useful additional sensitivity analysis to the standard inverse variance weighting (IVW) method (Burgess et al., 2017). Weighted medians can provide consistent estimates of causal effects, even if up to 50 per cent of the information in the analysis comes from the change of interest being an invalid instrumental variable. The weighted median approach has some important advantages over MR-Egger because it improves the accuracy of the results. When most instrumental variables with similar causal estimates are valid, the weighted model approach remains valid even if other instrumental variables do not satisfy the MR method’s requirements for causal inference (Bowden et al., 2016; Ooi et al., 2019; Chen et al., 2022).

#### Sensitivity analysis

To ensure the robustness of the results, a series of sensitivity analyses were performed. Cochran’s *Q* statistics were used to assess heterogeneity among IVs, concluding no heterogeneity when *p* > 0.05 (Huedo-Medina et al., 2006). Horizontal polytropic was tested using MR-Egger regression, examining the intercept term to evaluate its impact on the MR analysis results (Burgess and Thompson, 2017). Leave-one-out analysis was used to determine if any of the SNPs were driving the causal estimates. Finally, the MR Pleiotropy Residual Sum and Outlier (MR-PRESSO) test was employed to identify and correct for outliers in the IVW linear regression, eliminating SNPs associated with heterogeneity (Frazier and Price, 1998; Wu et al., 2020).

## Results

### Causal effects of gut microbiota on PM and DM

**Figure 2:**
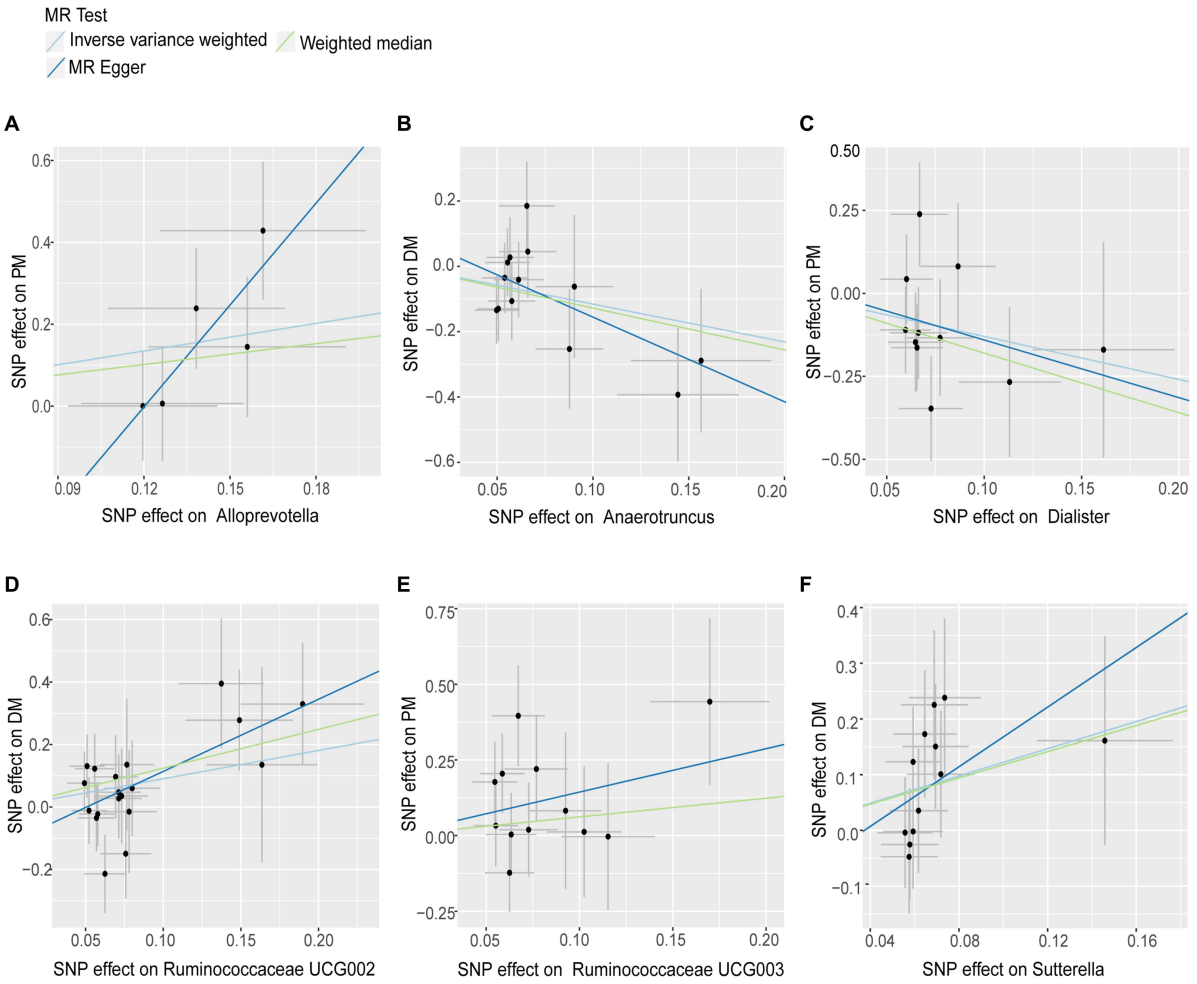
Scatter plots for Gut Microbiota on Polymyositis and Dermatomyositis. The pleiotropic effect was detected by testing whether the y-intercept from the MR-ER analysis was zero. Causal effects of **(A)** Alloprevotella, **(C)** Dialister and **(E)** Ruminococcaceae UCG003 on the risk of Polymyositis; Causal effects of (B) Anaerotruncus, **(D)** Ruminococcaceae UCG002 and **(F)** Sutterella on the risk of Dermatomyositis.

As shown in Table 1 and Figure 2, we observed a certain genus-level taxa of gut microbiota have causal effects on the risk of PM and DM. Specifically, a higher genetically predicted level of *Alloprevotella* (OR: 2.626, 95% CI: 1.095–6.298, *p* = 0.031) and *Ruminococcaceae UCG003* (OR: 4.219, 95% CI: 1.227–14.511, *p* = 0.022) was associated with an increased risk of PM, while *Dialister* was found to be related to a lower risk of PM (OR:0.273, 95% CI:0.077–0.974, *p* = 0.045). For DM, genus *Anaerotruncus* showed a negative correlation with DM (OR: 0.314, 95%CI: 0.112–0.882, *p* = 0.028), indicating a lower risk of DM, while *Ruminococcaceae UCG002* (OR:2.439, 95% CI:1.173–5.071, *p* = 0.017) and *Sutterella* (OR:3.392, 95% CI:1.302–8.839, *p* = 0.012) were associated with an increased risk of DM. Detailed information on the specific SNPs used in the analysis is provided in the Supplementary materials.

**Table 1:** Significant MR analysis results in the discovery of gut microbiota.

	N of SNPs	OR	Lower	Upper	*p-*value
**PM**					
***Alloprevotella***					
IVW	6	2.626	1.095	6.298	0.031
MR-Egger	6	1,608.028	0.502	5,150,966.673	0.147
WME	6	1.609	0.557	4.644	0.379
***Dialister***					
IVW	11	0.273	0.077	0.974	0.045
MR-Egger	11	0.176	0.001	41.131	0.548
WME	11	0.166	0.029	0.941	0.042
***Ruminococcaceae* UCG003**					
IVW	12	4.219	1.227	14.511	0.022
MR-Egger	12	4.189	0.075	235.050	0.502
WME	12	1.855	0.340	10.116	0.476
**DM**					
***Anaerotruncus***					
IVW	13	0.314	0.112	0.882	0.028
MR-Egger	13	0.075	0.004	1.526	0.120
WME	13	0.278	0.067	1.160	0.079
***Ruminococcaceae* UCG002**					
IVW	22	2.439	1.173	5.071	0.017
MR-Egger	22	8.456	1.223	58.478	0.043
WME	22	2.802	1.008	7.787	0.048
***Sutterella***					
IVW	12	3.392	1.302	8.839	0.012
MR-Egger	12	14.542	0.226	936.653	0.236
WME	12	3.245	0.886	11.889	0.076

N of SNP is the number of SNPs being used as IVs.

### Reverse Mendelian randomization analysis

In order to explore whether PM/DM had any causal effect on the identified important bacterial genera, we also performed a reverse MR analysis using SNPs associated with PM/DM as IVs (i.e., PM/DM as the exposure and the identified pathogenic bacterial genera as the outcome), and we did not find statistically significant associations between PM/DM and the identified pathogenic bacterial genera, as shown in Table 2. We performed a series of sensitivity analyses (same as MR analyses) to ensure the robustness of the results.

**Table 2:** Reverse MR analysis results between PM/DM and gut microbiota.

		N of SNPs	OR	Lower	Upper	*p*-value
**Exposure**	**Outcome**					
**PM**	***Alloprevotella***					
	IVW	2	0.985	0.929	1.044	0.623
	MR-Egger	2	/	/	/	/
	WME	2	/	/	/	/
	***Dialister***					
	IVW	8	1.007	0.993	1.021	0.310
	MR-Egger	8	1.024	0.984	1.065	0.293
	WME	8	1.016	0.998	1.034	0.079
	***Ruminococcaceae* UCG003**					
	IVW	8	0.995	0.983	1.007	0.413
	MR-Egger	8	0.992	0.956	1.023	0.678
	WME	8	0.993	0.977	1.009	0.390
						
**DM**	***Anaerotruncus***					
	IVW	6	1.001	0.963	1.041	0.938
	MR-Egger	6	1.116	0.745	1.672	0.623
	WME	6	1.005	0.976	1.034	0.758
	***Ruminococcaceae* UCG002**					
	IVW	6	1.008	0.987	1.029	0.464
	MR-Egger	6	1.094	0.894	1.339	0.431
	WME	6	0.997	0.969	1.027	0.851
	***Sutterella***					
	IVW	6	0.991	0.968	1.015	0.455
	MR-Egger	6	1.031	0.821	1.295	0.803
	WME	6	0.989	0.960	1.018	0.445

N of SNP is the number of SNPs being used as IVs.

### Sensitivity analysis

**Figure 3:**
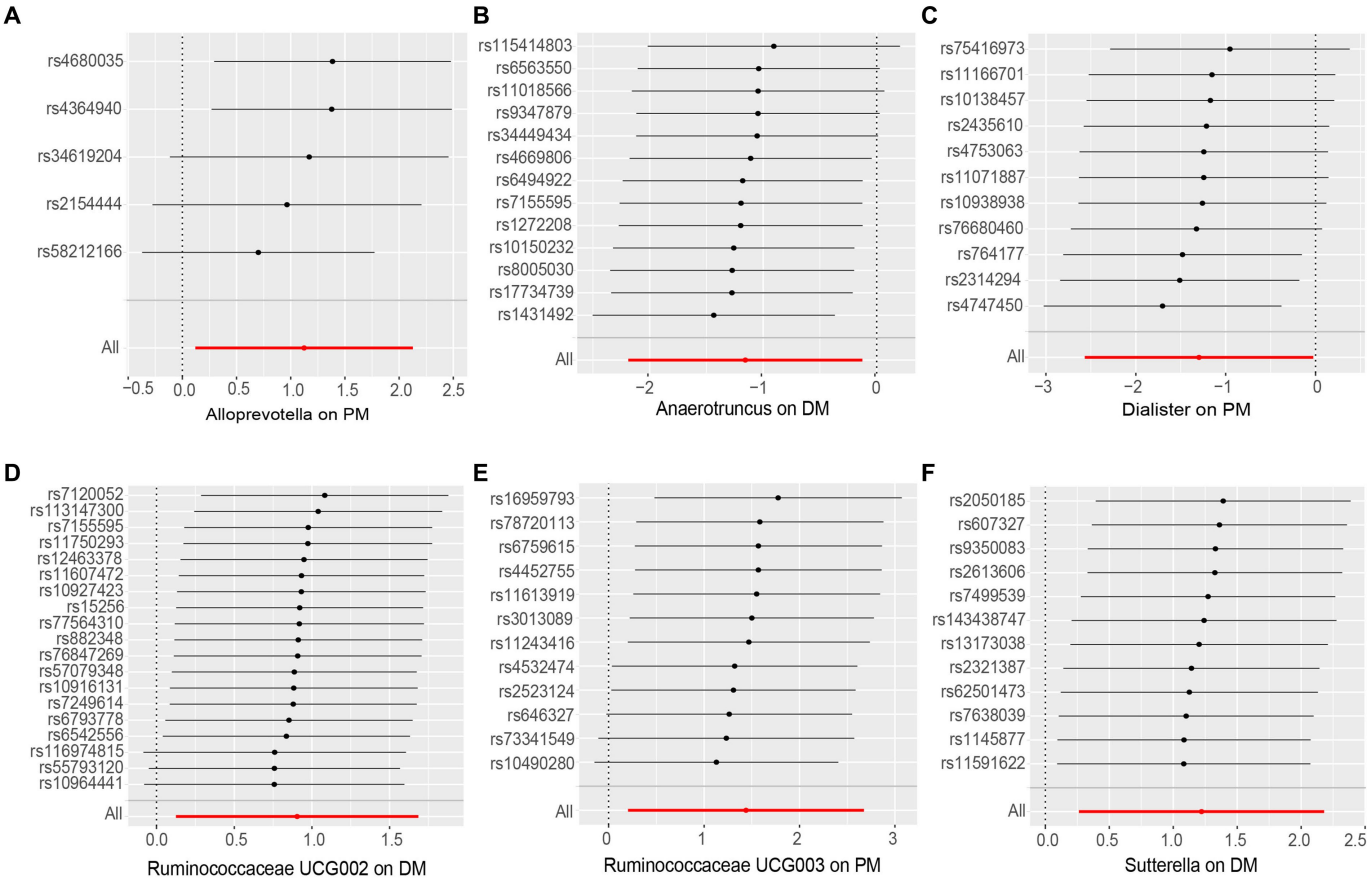
Leave-one-out analysis of the causal effect of Gut Microbiota on PM and DM. **(A)** Alloprevotella, **(B)** Anaerotruncus, **(C)** Dialister, **(D)** Ruminococcaceae UCG002, **(E)** Ruminococcaceae UCG003 and **(F)** Sutterella. Red lines represent estimations from the IVW test.

None of the MR-Egger regression intercepts deviated from zero, indicating no evidence of horizontal pleiotropy (all intercepts *p* > 0.05). Additionally, the leave-one-out analysis demonstrated consistent causal estimations, suggesting that none of the identified causal associations were heavily driven by any single IV. The results of the sensitivity analysis are presented in Table 3 and Figure 3.

**Table 3:** Correlation of heterogeneity and pleiotropy tests for polymyositis and dermatomyositis with genetic predictors of gut microbiota.

	Q-value (IVW)	P Q (IVW)	Q-value (MR-ER)	PQ (MR-ER)	Intercept	P Intercept
**PM**						
*Alloprevotella*	5.103	0.404	2.646	0.619	−0.900	0.192
*Dialister*	9.775	0.460	9.746	0.371	0.033	0.874
*Ruminococcaceae* UCG003	9.929	0.537	9.929	0.447	0.001	0.997
**DM**						
*Anaerotruncus*	9.067	0.697	8.083	0.7.59	0.100	0.342
*Ruminococcaceae* UCG002	13.660	0.884	11.810	0.923	−0.100	0.189
*Sutterella*	6.992	0.805	6.427	0.778	−0.100	0.498

## Discussion

This study represents the first attempt to investigate the causal relationship between gut microbiota and PM/DM using a two-sample MR analysis. Our findings reveal five different bacterial genera (*Alloprevotella, Ruminococcaceae, Anaerotruncus, Sutterella*, and *Dialister*) that are associated with PM and DM. Notably, the genus *Ruminococcaceae* was found to be causally involved in both conditions, which may allow us to early prevention strategies rather than just treating the disease after it has progressed (Xi et al., 2023). It is also worth noting that muscle biopsy is invasive and requires a definitive diagnosis of PM/DM (Findlay et al., 2015), and that non-invasive, convenient, and rapid fecal testing for intestinal flora offers excellent opportunities.

Gut microbes associated with PM and DM play an important role in prevention and treatment. One particular bacterium we studied, *Ruminococcaceae* (De Weirdt and Van de Wiele, 2015), belonging to the Clostridium sp. *Blautia* genus in the *Lachnospiraceae* family (Azzouz et al., 2019). It is a strictly anaerobic bacterium known for its ability to promote intestinal health by producing butyrate and other short-chain fatty acids (SCFA) (Gu et al., 2022). *Ruminococcaceae* is naturally present in the colonic mucosa of healthy individuals and has been found to play an important role in the development of various autoimmune diseases such as ankylosing spondylitis, lupus, and inflammatory bowel disease (Azzouz et al., 2019; Zhang et al., 2021). Recent reports have shown that *Ruminococcaceae* is present in high abundance in patients with these conditions.

Through our research, we discovered that the relative abundance of *Ruminococcaceae* may be related to disease activity in PM as well as DM. Although *Ruminococcaceae* are commonly found in low levels in the feces of healthy populations (Liu et al., 2008), even patients with mild disease activity showed higher-than-normal levels of *Ruminococcaceae*. We observed that patients with high disease activity had even higher levels of *Ruminococcaceae*, leading us to hypothesize that increased activity of this bacterium could raise the risk of developing PM/DM. Considering this association, monitoring the abundance of *Rumenococcaceae* flora at an early stage can provide targeted recommendations for preventive and therapeutic approaches.

Several studies have indicated that butyrate-producing bacteria promote the development of regulatory T cells (Tregs) in the gut-associated lymphatic system, which helps restore immune homeostasis and reduce the risk of autoimmune disease pathogenesis (Chen et al., 2020; Zhufeng et al., 2022). Additionally, butyrate production by *Ruminococcaceae* is known to enhance gut health, support epithelial cell function and morphology, and regulate the balance of intestinal flora (Zhufeng et al., 2022).

However, it is important to note that butyrate production by *Ruminococcaceae* inhibits histone deacetylase (HDAC), which can affect the accumulation of immune-related molecules and CD8+ T cells. The increase in CD8+ T cells has been associated with muscle fiber destruction observed in PM, suggesting a potential mechanism linking *Ruminococcaceae* and an increased risk of PM. Furthermore, evidence from observational studies, MR analyses, and clinical trials suggests that the effects of *Ruminococcaceae* may vary depending on the specific species and strain. Genomic analysis has revealed that certain *Ruminococcaceae* strains associated with autoimmune diseases are distinct from those found in healthy individuals (Azzouz et al., 2019). However, further investigation at a specialized genomic level is necessary to fully understand this new finding.

Overall, the interactions between *Ruminococcaceae* and autoimmune diseases like PM involve butyrate production, modulation of Tregs, influence on immune-related molecules and CD8+ T cells, and strain-specific determinants that require in-depth genomic analysis for better insights.

The strength of this study lies in the early monitoring of risk factors and the availability of effective treatment strategies that can minimize the burden of healthcare costs and disease suffering. Our study utilized MR to use genetic letters as IVs, thereby predicting the relationship between gut flora and PM/DM at the genetic level (Xi et al., 2023). The strengths of our study compared with previous studies are threefold: first, it is the first attempt of its kind, giving us more possibilities to study this type of disease; second, it lies in our rigorous selection of IVs and thorough sensitivity analyses to ensure the validity of our causal estimates. During the IV selection process, we employed stringent quality control measures, including the use of independent GW AS SNPs and the assessment of horizontal pleiotropy; finally, the large sample size of this study and the European origin of the participants further enhanced the reliability of our results. These advantages help to deepen our understanding of the relationship between gut flora and PM/DM and facilitate the development of therapeutic strategies for PM/DM.

Indeed, certain limitations in our study should be acknowledged. Firstly, our analysis of the intestinal flora was conducted at the genus level, and a more detailed analysis at the species or strain level could provide deeper insights and improve the accuracy of the findings. By examining specific bacterial species or strains, we could potentially identify more precise associations with PM and DM. Additionally, it is important to consider the age differences in the occurrence of PM and DM. DM shows a bimodal pattern of incidence, with peaks in childhood and later between the ages of 50 and 70 years. On the other hand, PM is rare in childhood and typically occurs after the second decade of life. Furthermore, both conditions are more common in females (Dalakas and Hohlfeld, 2003; Khan and Christopher-Stine, 2011). Gender differences are known to exist in autoimmune disorders, but our study did not explore the gender-specific effects of the gut microbiota on PM and DM. Future research should aim to investigate these potential differences and explore the specific associations between gut microbiota, gender, and PM/DM. Another aspect to be considered is the threshold used for statistical significance. In our study, we employed a *p*-value threshold of *p* < 1 × 10^−5^, which may have resulted in some significant associations being missed. Future studies aiming to establish a causal relationship between gut bacteria and PM/DM should consider using a more stringent threshold to ensure robust findings.

Moving forward, it is important for future studies to address these limitations. More detailed analysis at the species or strain level, consideration of age and gender differences, and the use of appropriate statistical thresholds will enable a more comprehensive understanding of the relationship between gut microbiota and PM/DM and provide more reliable strategies for early monitoring and treatment.

In Summary, our study reveals the causal impact of specific gut microbiota on PM/DM. This finding opens up new avenues for future studies of these diseases. In particular, our finding of a consistent association between *Ruminococcaceae* and PM and DM could provide a promising direction for early prevention of the disease. These findings contribute to a better understanding of the intricate relationship between the gut microbiota and PM/DM. In addition, more in-depth investigations at a finer taxonomic level and consideration of the influence of gender will enhance our knowledge and potentially lead to the study of therapeutic interventions targeting the gut microbiota for the effective management of these diseases. It is important to note that these findings are based on the current scientific evidence and the interpretation of the study results. For these individuals with abnormal intestinal flora, it is important to consider early and aggressive correction of intestinal dysbiosis, which can be done through dietary changes, probiotic supplementation, and increased physical activity. Especially for patients with dysbiosis of the *Ruminococcaceae*, direct oral administration of probiotics may be considered. Precise personalized treatments, such as individually testing the individual gut microbiota, designing probiotic strains that are relevant to a particular individual person, dietary changes, and administration of specific probiotic preparations may help to improve muscle inflammatory responses and symptoms and improve the quality of life of patients with PM/DM in the early stages of the disease, leading to secondary prevention of the disease. As research progresses, new findings and insights may further shape our understanding of these diseases and their relationship with gut microbiota. Consulting with medical professionals and experts in the field can provide personalized guidance and recommendations for individuals experiencing or at risk of developing polymyositis and dermatomyositis.

## Data Availability

The datasets presented in this study can be found in online repositories. The names of the repository/repositories and accession number(s) can be found in the article/Supplementary material.

## Footnotes

^1^ http://www.phenoscanner.medschl.cam.ac.uk

## Glossary

PM: polymyositis
DM: dermatomyositis
GWAS: Genome Wide Association Studies
MR: Mendelian randomization
IVW: inverse-variance weighted
IIM: idiopathic inflammatory myopathies
FC: Firmicutes
BT: Bacteroidetes
IBD: inflammatory bowel disease
SNPs: Single nucleotide polymorphisms
IVs: instrumental variables
DAG: Directed acyclic graph
WME: weighted median
MR-PRESSO: MR Pleiotropy Residual Sum and Outlier
OR: odds ratio
Q-value: statistics of Cochrane’s Q test
PQ: value corresponding to Cochrane’s Q test
P intercept: *p*-value corresponding to MR-ER intercept test
MR-ER: Mendelian randomization-Egger regression
HDAC: inhibits histone deacetylase
