# Supplementary material for: Genetically predicted the causal relationship between gut microbiota and the risk of polymyositis/dermatomyositis: a Mendelian randomization analysis
Source: Front Microbiol. 2024 Aug 21;15:1409497. doi: 10.3389/fmicb.2024.1409497 (PMC11371719; doi:10.3389/fmicb.2024.1409497)
Supplement: Supplementary file 1 [file Table_1.DOCX]

**Supplemental Table 1:** Summary of the 5 SNPs associated with PM and *Alloprevotella*; PM, Polymyositis.

Effect on *Alloprevotella* Effect on PM

| SNP | Chromosome | Position | Effect allele | Other allele | Beta | SE | P value | Beta | SE | P value |
| --- | --- | --- | --- | --- | --- | --- | --- | --- | --- | --- |
| rs2154444 | 21 | 36053643 | G | T | -0.138234 | 0.0308961 | 8.37E-06 | -0.2388 | 0.1471 | 0.1044 |
| rs34619204 | 21 | 40993008 | G | A | -0.156037 | 0.0344392 | 8.84E-06 | -0.1449 | 0.1712 | 0.3973 |
| rs4364940 | 1 | 234126516 | A | G | 0.126418 | 0.0282207 | 8.58E-06 | 0.0065 | 0.1397 | 0.9631 |
| rs4680035 | 3 | 152805600 | G | A | 0.119599 | 0.0259446 | 4.99E-06 | 4.00E-04 | 0.133 | 0.9974 |
| rs58212166 | 4 | 189076705 | A | G | -0.161506 | 0.0359102 | 7.94E-06 | -0.4286 | 0.1682 | 0.0108101 |

**Supplemental Table 2:** Summary of the 11 SNPs associated with PM and *Dialister*; PM, Polymyositis.

Effect on *Dialister* Effect on PM

| SNP | Chromosome | Position | Effect allele | Other allele | Beta | SE | P value | Beta | SE | P value |
| --- | --- | --- | --- | --- | --- | --- | --- | --- | --- | --- |
| rs10138457 | 14 | 102458052 | T | C | -0.113054 | 0.0261933 | 7.88E-06 | 0.267 | 0.2247 | 0.2347 |
| rs10938938 | 4 | 23294359 | A | G | 0.0773514 | 0.0170915 | 7.37E-06 | -0.1339 | 0.1758 | 0.4465 |
| rs11071887 | 15 | 32955095 | T | C | 0.0662403 | 0.0146315 | 5.91E-06 | -0.1188 | 0.1385 | 0.391 |
| rs11166701 | 8 | 138295725 | G | A | -0.0655301 | 0.013186 | 5.51E-07 | 0.1636 | 0.1296 | 0.2067 |
| rs2314294 | 16 | 9336862 | T | C | 0.0865932 | 0.0193719 | 8.08E-06 | 0.0813 | 0.1898 | 0.668299 |
| rs2435610 | 7 | 150890034 | A | C | 0.0647132 | 0.0143314 | 5.93E-06 | -0.147 | 0.1497 | 0.3261 |
| rs4747450 | 10 | 23199653 | C | A | 0.0668509 | 0.0147685 | 5.84E-06 | 0.2385 | 0.1562 | 0.1268 |
| rs4753063 | 11 | 92267129 | A | G | 0.0596297 | 0.0130052 | 4.86E-06 | -0.1102 | 0.1301 | 0.3971 |
| rs75416973 | 1 | 172809633 | A | G | 0.0727223 | 0.016451 | 9.46E-06 | -0.347 | 0.157 | 0.0270901 |
| rs764177 | 3 | 45809449 | C | A | -0.0601454 | 0.0135366 | 9.61E-06 | -0.043 | 0.1348 | 0.749701 |
| rs76680460 | 9 | 25447080 | G | A | -0.161296 | 0.0364332 | 8.19E-06 | 0.1697 | 0.3251 | 0.6018 |

**Supplemental Table 3:** Summary of the 12 SNPs associated with PM and *Ruminococcaceae* UCG003; PM, Polymyositis.

Effect on *Ruminococcaceae* UCG003 Effect on PM

| SNP | Chromosome | Position | Effect allele | Other allele | Beta | SE | P value | Beta | SE | P value |
| --- | --- | --- | --- | --- | --- | --- | --- | --- | --- | --- |
| rs10490280 | 2 | 37905976 | C | T | -0.0672094 | 0.0143309 | 4.16E-06 | -0.396 | 0.1659 | 0.0170098 |
| rs11243416 | 9 | 134416970 | T | C | -0.0925653 | 0.0191203 | 1.67E-06 | -0.0822 | 0.2585 | 0.750501 |
| rs11613919 | 12 | 75496463 | G | T | 0.0727604 | 0.0155716 | 1.63E-06 | 0.0195 | 0.1556 | 0.9003 |
| rs16959793 | 15 | 35071718 | A | C | -0.062527 | 0.0131259 | 2.22E-06 | 0.1228 | 0.1314 | 0.3503 |
| rs2523124 | 7 | 97348436 | C | T | 0.0546625 | 0.0120823 | 5.78E-06 | 0.1773 | 0.132 | 0.1793 |
| rs3013089 | 1 | 13794594 | G | A | -0.0551473 | 0.0120346 | 4.38E-06 | -0.0329 | 0.1338 | 0.806 |
| rs4452755 | 8 | 82026852 | A | C | -0.0634463 | 0.0134741 | 3.29E-06 | -0.0037 | 0.1367 | 0.9784 |
| rs4532474 | 6 | 105781538 | G | A | 0.0769231 | 0.017045 | 4.82E-06 | 0.2202 | 0.1745 | 0.2071 |
| rs646327 | 19 | 49209851 | G | A | 0.0586696 | 0.0118369 | 7.83E-07 | 0.2047 | 0.1314 | 0.1193 |
| rs6759615 | 2 | 205238716 | A | G | 0.102523 | 0.0200204 | 7.86E-07 | 0.0124 | 0.216 | 0.9541 |

rs73341549 7 51541468 T C -0.169857 0.0318768 1.51E-07 -0.4426 0.2761 0.109

rs78720113 3 41982393 A G -0.115348 0.0249601 7.59E-06 0.0032 0.2428 0.9895

**Supplemental Table 4:** Summary of the 13 SNPs associated with DM and *Anaerotruncus*; DM, Dermatomyositis.

Effect on *Anaerotruncus* Effect on DM

| SNP | Chromosome | Position | Effect allele | Other allele | Beta | SE | P value | Beta | SE | P value |
| --- | --- | --- | --- | --- | --- | --- | --- | --- | --- | --- |
| rs10150232 | 14 | 30418008 | A | G | 0.0567088 | 0.0124877 | 6.68E-06 | 0.0272 | 0.1232 | 0.825 |
| rs11018566 | 11 | 89040226 | A | G | -0.156465 | 0.0366032 | 6.14E-06 | 0.2888 | 0.2197 | 0.1886 |
| rs115414803 | 4 | 88163243 | A | C | -0.144356 | 0.0317524 | 6.83E-06 | 0.393 | 0.2043 | 0.0543901 |
| rs1272208 | 9 | 78630894 | T | G | 0.0611743 | 0.0129831 | 4.28E-06 | -0.041 | 0.1157 | 0.723 |
| rs1431492 | 3 | 150855371 | C | T | -0.0654996 | 0.0146188 | 7.36E-06 | -0.1849 | 0.1349 | 0.1704 |
| rs17734739 | 2 | 211663702 | T | C | 0.0660052 | 0.014908 | 7.43E-06 | 0.045 | 0.1415 | 0.750501 |
| rs34449434 | 12 | 76523655 | A | C | -0.0497004 | 0.0113402 | 9.85E-06 | 0.1342 | 0.104 | 0.1969 |
| rs4669806 | 2 | 12200752 | G | T | 0.0576389 | 0.0122994 | 2.42E-06 | -0.1064 | 0.12 | 0.3752 |
| rs6494922 | 15 | 33459867 | A | G | 0.0903106 | 0.0202257 | 6.62E-06 | -0.0622 | 0.2187 | 0.776 |
| rs6563550 | 13 | 38058413 | T | C | 0.0877135 | 0.0176745 | 2.35E-07 | -0.253 | 0.1825 | 0.1657 |
| rs7155595 | 14 | 77502546 | C | A | 0.0539336 | 0.0118903 | 7.55E-06 | -0.0352 | 0.1072 | 0.7424 |

rs8005030 14 30607199 C T 0.0554447 0.0117854 2.28E-06 0.0117 0.1044 0.9105

rs9347879 6 165015261 T C 0.050618 0.011049 4.22E-06 -0.13 0.0984 0.1865

**Supplemental Table 5:** Summary of the 19 SNPs associated with DM and *Ruminococcaceae* UCG002; DM, Dermatomyositis.

Effect on *Ruminococcaceae* UCG002 Effect on DM

| SNP | Chromosome | Position | Effect allele | Other allele | Beta | SE | P value | Beta | SE | P value |
| --- | --- | --- | --- | --- | --- | --- | --- | --- | --- | --- |
| rs10916131 | 1 | 227563126 | C | T | -0.069333 | 0.0146753 | 2.87E-06 | -0.0966 | 0.1329 | 0.4673 |
| rs10927423 | 1 | 14732458 | C | A | -0.0713617 | 0.0147712 | 8.50E-07 | -0.0275 | 0.1281 | 0.8299 |
| rs10964441 | 9 | 20131746 | G | A | -0.14906 | 0.034486 | 7.45E-06 | -0.2779 | 0.1626 | 0.0875609 |
| rs113147300 | 9 | 114066670 | A | G | -0.075842 | 0.0164562 | 7.69E-06 | 0.1495 | 0.1423 | 0.2933 |
| rs11607472 | 11 | 43344751 | A | G | -0.0780229 | 0.0176325 | 7.19E-06 | 0.0149 | 0.1971 | 0.9396 |
| rs116974815 | 11 | 111712942 | C | A | -0.189731 | 0.0396566 | 2.03E-06 | -0.3295 | 0.1961 | 0.0929908 |
| rs11750293 | 5 | 123822114 | G | T | -0.0578304 | 0.0120512 | 1.76E-06 | 0.0226 | 0.1023 | 0.8255 |
| rs12463378 | 19 | 54475808 | A | G | -0.0522061 | 0.0112086 | 2.96E-06 | 0.0116 | 0.1073 | 0.9138 |
| rs15256 | 10 | 73820548 | C | T | 0.0732376 | 0.016834 | 9.46E-06 | 0.0353 | 0.1515 | 0.8158 |
| rs55793120 | 12 | 47384118 | T | C | 0.137396 | 0.027414 | 4.81E-07 | 0.3951 | 0.2078 | 0.05719 |
| rs57079348 | 13 | 87920450 | T | G | -0.0765729 | 0.0172819 | 7.22E-06 | -0.1355 | 0.2112 | 0.5212 |

| rs6542556 | 2 | 120757853 | G | A | -0.050974 | 0.011406 | 7.86E-06 | -0.1309 | 0.1014 | 0.1968 |
| --- | --- | --- | --- | --- | --- | --- | --- | --- | --- | --- |
| rs6793778 | 3 | 24045453 | T | C | 0.0558706 | 0.0125258 | 9.81E-06 | 0.1226 | 0.1123 | 0.2748 |
| rs7120052 | 11 | 86335459 | A | C | 0.0624796 | 0.0135523 | 1.97E-06 | -0.2138 | 0.1252 | 0.087791 |
| rs7155595 | 14 | 77502546 | C | A | 0.0569929 | 0.0116986 | 1.15E-06 | -0.0352 | 0.1072 | 0.7424 |
| rs7249614 | 19 | 43637890 | G | A | 0.0492778 | 0.0110807 | 9.07E-06 | 0.0764 | 0.1011 | 0.4499 |
| rs76847269 | 5 | 141014951 | A | G | 0.163508 | 0.0356151 | 5.17E-06 | 0.1352 | 0.3121 | 0.664899 |
| rs77564310 | 15 | 80713184 | A | C | -0.0713093 | 0.0140855 | 3.29E-07 | -0.0472 | 0.1217 | 0.6979 |
| rs882348 | 4 | 23325040 | A | G | -0.0799946 | 0.0178618 | 5.45E-06 | -0.0599 | 0.1527 | 0.6947 |

**Supplemental Table 6:** Summary of the 12 SNPs associated with DM and *Sutterella*; DM, Dermatomyositis.

Effect on *Sutterella* Effect on DM

| SNP | Chromosome | Position | Effect allele | Other allele | Beta | SE | P value | Beta | SE | P value |
| --- | --- | --- | --- | --- | --- | --- | --- | --- | --- | --- |
| rs1145877 | 6 | 82386821 | G | A | 0.073548 | 0.0162413 | 7.20E-06 | 0.2382 | 0.1424 | 0.09438 |
| rs11591622 | 10 | 102520687 | T | G | -0.0688382 | 0.0151375 | 6.50E-06 | -0.2254 | 0.1336 | 0.0915103 |
| rs13173038 | 5 | 58499183 | A | G | -0.0718076 | 0.0151627 | 2.73E-06 | -0.1008 | 0.114 | 0.3764 |
| rs143438747 | 1 | 63278549 | T | C | -0.145793 | 0.0306867 | 3.28E-06 | -0.1611 | 0.1876 | 0.3906 |
| rs2050185 | 6 | 147936781 | A | G | -0.0575138 | 0.0128766 | 7.97E-06 | 0.0475 | 0.1017 | 0.6402 |
| rs2321387 | 13 | 58689340 | G | A | -0.0592876 | 0.0124509 | 1.87E-06 | -0.123 | 0.0995 | 0.2162 |
| rs2613606 | 7 | 111285025 | T | C | 0.0556794 | 0.0124117 | 7.20E-06 | -0.004 | 0.0996 | 0.9676 |
| rs607327 | 11 | 111695709 | T | C | -0.0578334 | 0.0129053 | 6.63E-06 | 0.0255 | 0.1014 | 0.8018 |
| rs62501473 | 7 | 150126751 | G | A | 0.0694233 | 0.0149416 | 5.52E-06 | 0.1505 | 0.1121 | 0.1794 |
| rs7499539 | 16 | 85038065 | A | G | 0.0617478 | 0.0130998 | 2.36E-06 | 0.0354 | 0.1116 | 0.751201 |
| rs7638039 | 3 | 70588939 | T | C | 0.0645624 | 0.0143887 | 8.66E-06 | 0.1729 | 0.1142 | 0.1299 |

rs9350083 6 18583878 T G -0.0593069 0.0133929 8.23E-06 0.002 0.1027 0.9846
