# Supplementary material for: Genetically predicted the causal relationship between gut microbiota and the risk of polymyositis/dermatomyositis: a Mendelian randomization analysis
Source: Front Microbiol. 2024 Aug 21;15:1409497. doi: 10.3389/fmicb.2024.1409497 (PMC11371719; doi:10.3389/fmicb.2024.1409497)
Supplement: Supplementary file 2 [file Data_Sheet_1.docx]

**STROBE-MR checklist of recommended items to address in reports of Mendelian randomization studies**^1^ ^2^

| **Item No.** | **Section** | **Checklist item** | **Relevant text from manuscript** |
| --- | --- | --- | --- |
| 1 | **TITLE and ABSTRACT** | Indicate Mendelian randomization (MR) as the study’s design in the title and/or the abstract if that is a main purpose of the study | Genetically Predicted the Causal Relationship Between Gut Microbiota and the Risk of Polymyositis/Dermatomyositis: A Mendelian Randomization Analysis |
|  | **INTRODUCTION** |  |  |
| 2 | **Background** | Explain the scientific background and rationale for the reported study. What is the exposure? Is a potential causal relationship between exposure and outcome plausible? Justify why MR is a helpful method to address the study question | To address the need for causal inference and identify the primary influencing factors within the gut microbiome, we conducted a Mendelian randomization (MR) analysis. MR analysis was employed as a driving force for further research to meet the need for causal inference and to identify key factors influencing the gut microbiome. By utilizing genetic instruments to minimize confounding factors, MR analysis enables the assessment of causal effects and aims to uncover the potential relationship between gut microbiota and Polymyositis (PM) and dermatomyositis (DM). This analysis furthers understanding of the role of gut flora in PM/DM by identifying relevant gut microbes to facilitate early disease screening, implementation of interventions, and personalized healthcare. Previous observational studies have emphasized the link between reduced microbial diversity and DM, but a causal relationship has not been established. In addition, there is a dearth of research on the key gut microbiota that may impact the development of PM. This study aims to elucidate the specific gut microflora that potentially contribute to the onset of PM/DM and assess their potential as novel targets for treatment. |
| 3 | **Objectives** | State specific objectives clearly, including pre-specified causal hypotheses (if any). State that MR is a method that, under specific assumptions, intends to estimate causal effects | MR analysis enables the assessment of causal effects and aims to uncover the potential relationship between gut microbiota and Polymyositis (PM) and dermatomyositis (DM). |
|  | **METHODS** |  |  |
| 4 | **Study design and data sources** | Present key elements of the study design early in the article. Consider including a table listing sources of data for all phases of the study. For each data source contributing to the analysis, describe the following: | This study employs a two-sample Mendelian randomization (MR) analysis, utilizing summary statistics from Genome Wide Association Studies (GWAS), to explore the potential causal effect of gut microbiota on PM and DM. Single nucleotide polymorphisms (SNPs) significantly associated with the gut microbiota were selected as instrumental variables (IVs), and three assumptions were made to ensure accuracy. First, the genetic variants selected as IVs should be significantly associated with gut microbiota. Second, IVs should not be associated with any confounding factors. Third, IVs should affect the risk of PM and DM only through gut microbiota. A series of sensitivity analyses were then conducted for significant associations.  Summary statistics of the gut microbiota were obtained from a large-scale multi-ethnic GWAS meta-analysis, involving 18340 individuals from 24 cohorts, most of which were of European ancestry. Genus-level taxa were analyzed, with a total of 131 genera showing an average abundance greater than 1%. However, 12 of these genera were unknown, leaving 119 genus-level taxa included in this study. Covariate adjustments were made for gender, age, technical covariates, and genetic principal components using Spearman correlation analysis.  The outcome datasets were extracted from the large publicly available GWAS analysis. Summary data for DM were obtained from a GWAS study of European ancestry, including 208 cases and 213,145 controls. For polymyositis (PM), the study included 119 cases and 213,145 controls. |
|  | a) | Setting: Describe the study design and the underlying population, if possible. Describe the setting, locations, and relevant dates, including periods of recruitment, exposure, follow-up, and data collection, when available. | Prior to the MR analysis, a rigorous screening process was conducted to ensure the reliability of the SNPs and meet the assumptions of MR analysis. The IVs were selected based on SNPs associated with gut microbiota at a genome-wide significance level (p<1 × 10^-5^). A linkage disequilibrium (LD) aggregation threshold of r2 < 0.001 was applied to remove correlated and dependent SNPs to ensure independence and validity. To assess potential confounding effects, the selected SNPs were evaluated for associations with other phenotypes using the publicly available PhenoScanner V2 (http://www.phenoscanner. medschl.cam.ac.uk). SNPs related to any potential confounders were removed at genome-wide significance. Additionally, only SNPs with consistent effect alleles between the exposure and outcome GWAS datasets were included, while those absent in the outcome GWAS were removed. In addition, to eliminate the bias of weak IVs, we calculated the F-statistic for SNPs using the following formula: F = r2(n-k-1)/[k(1-r2)]. the value of the F-statistic indicates the strength of the IV, and F-statistic > 10 is a strong IV. |
|  | b) | Participants: Give the eligibility criteria, and the sources and methods of selection of participants. Report the sample size, and whether any power or sample size calculations were carried out prior to the main analysis | Genus-level taxa were analyzed, with a total of 131 genera showing an average abundance greater than 1%. However, 12 of these genera were unknown, leaving 119 genus-level taxa included in this study. Covariate adjustments were made for gender, age, technical covariates, and genetic principal components using Spearman correlation analysis.  Summary data for DM were obtained from a GW AS study of European ancestry, including 208 cases and 213,145 controls. For polymyositis (PM), the study included 119 cases and 213,145 controls. |
|  | c) | Describe measurement, quality control and selection of genetic variants | Prior to the MR analysis, a rigorous screening process was conducted to ensure the reliability of the SNPs and meet the assumptions of MR analysis. The IVs were selected based on SNPs associated with gut microbiota at a genome-wide significance level (p<1 × 10^-5^). A linkage disequilibrium (LD) aggregation threshold of r2 < 0.001 was applied to remove correlated and dependent SNPs to ensure independence and validity. To assess potential confounding effects, the selected SNPs were evaluated for associations with other phenotypes using the publicly available PhenoScanner V2 (http://www.phenoscanner. medschl.cam.ac.uk). SNPs related to any potential confounders were removed at genome-wide significance. Additionally, only SNPs with consistent effect alleles between the exposure and outcome GWAS datasets were included, while those absent in the outcome GWAS were removed. In addition, to eliminate the bias of weak IVs, we calculated the F-statistic for SNPs using the following formula: F = r2(n-k-1)/[k(1-r2)]. the value of the F-statistic indicates the strength of the IV, and F-statistic > 10 is a strong IV. |
|  | d) | For each exposure, outcome, and other relevant variables, describe methods of assessment and diagnostic criteria for diseases | To assess potential confounding effects, the selected SNPs were evaluated for associations with other phenotypes using the publicly available PhenoScanner V2 (http://www.phenoscanner. medschl.cam.ac.uk)[19]. SNPs related to any potential confounders were removed at genome-wide significance. Additionally, only SNPs with consistent effect alleles between the exposure and outcome GWAS datasets were included, while those absent in the outcome GWAS were removed. |
|  | e) | Provide details of ethics committee approval and participant informed consent, if relevant | Summary statistics of the gut microbiota were obtained from a large-scale multi-ethnic GWAS meta-analysis, involving 18340 individuals from 24 cohorts, most of which were of European ancestry. |
| 5 | **Assumptions** | Explicitly state the three core IV assumptions for the main analysis (relevance, independence and exclusion restriction) as well assumptions for any additional or sensitivity analysis | This study employs a two-sample Mendelian randomization (MR) analysis, utilizing summary statistics from Genome Wide Association Studies (GWAS), to explore the potential causal effect of gut microbiota on PM and DM. Single nucleotide polymorphisms (SNPs) significantly associated with the gut microbiota were selected as instrumental variables (IVs), and three assumptions were made to ensure accuracy. First, the genetic variants selected as IVs should be significantly associated with gut microbiota. Second, IVs should not be associated with any confounding factors. Third, IVs should affect the risk of PM and DM only through gut microbiota. A series of sensitivity analyses were then conducted for significant associations. The overall flowchart of the study is presented in **Figure1**.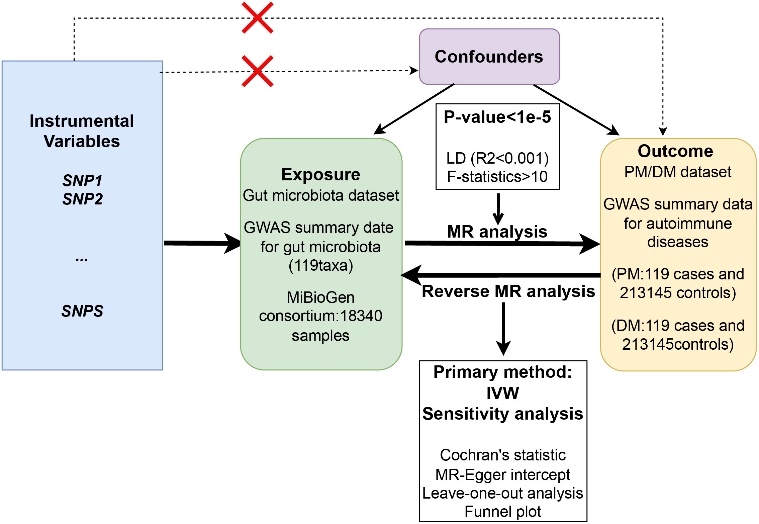 |
| 6 | **Statistical methods: main analysis** | Describe statistical methods and statistics used | To assess the causal relationship between gut microflora and outcomes, MR analysis was conducted using three methods: inverse variance weighting (IVW), MR-Egger regression, and weighted median (WME). These methods have been extensively described in previous studies. IVW analysis wasconsidered the most reliable method for estimating the causal relationship between exposure and outcome while being sensitive to pleiotropy. MR-Egger regression and WME were further applied as complementary analyses. MR-Egger regression helps detect potential bias in meta-analyses and assesses bias in polymorphism. WME provides consistent effect estimates in MR analyses even though up to half of the information comes from invalid IVs. |
|  | a) | Describe how quantitative variables were handled in the analyses (i.e., scale, units, model) | To assess the causal relationship between gut microflora and outcomes, MR analysis was conducted using three methods: inverse variance weighting (IVW), MR-Egger regression, and weighted median (WME). These methods have been extensively described in previous studies. |
|  | b) | Describe how genetic variants were handled in the analyses and, if applicable, how their weights were selected | As shown in Table 1 and Fig 3, we observed a certain genus-level taxa of gut microbiota have causal effects on the risk of PM and DM. Specifically, a higher genetically predicted level of Alloprevotella (OR: 2.626, 95% CI: 1.095-6.298, P=0.031) and Ruminococcaceae UCG003 (OR: 4.219, 95% CI: 1.227-14.511, P=0.022) was associated with an increased risk of PM, while Dialister was found to be related to a lower risk of PM (OR:0.273, 95% CI:0.077-0.974, P=0.045). For DM, genus Anaerotruncus showed a negative correlation with DM (OR: 0.314, 95%CI: 0.112-0.882, P=0.028), indicating a lower risk of DM, while Ruminococcaceae UCG002 (OR:2.439, 95% CI:1.173-5.071, P=0.017) and Sutterella (OR:3.392, 95% CI:1.302-8.839, P=0.012) were associated with an increased risk of DM. Detailed information on the specific SNPs used in the analysis is provided in the Supplement materials. |
|  | c) | Describe the MR estimator (e.g. two-stage least squares, Wald ratio) and related statistics. Detail the included covariates and, in case of two-sample MR, whether the same covariate set was used for adjustment in the two samples | To ensure the robustness of the results, a series of sensitivity analyses were performed. Cochran's Q statistics were used to assess heterogeneity among IVs, concluding no heterogeneity when P>0.05.  Detailed information on the specific SNPs used in the analysis is provided in the Supplement materials. |
|  | d) | Explain how missing data were addressed | Don not miss data |
|  | e) | If applicable, indicate how multiple testing was addressed | To assess potential confounding effects, the selected SNPs were evaluated for associations with other phenotypes using the publicly available PhenoScanner V2 (http://www.phenoscanner. medschl.cam.ac.uk). SNPs related to any potential confounders were removed at genome-wide significance. Additionally, only SNPs with consistent effect alleles between the exposure and outcome GWAS datasets were included, while those absent in the outcome GWAS were removed. In addition, to eliminate the bias of weak IVs, we calculated the F-statistic for SNPs using the following formula: F = r2(n-k-1)/[k(1-r2)]. the value of the F-statistic indicates the strength of the IV, and F-statistic > 10 is a strong IV. |
| 7 | **Assessment of assumptions** | Describe any methods or prior knowledge used to assess the assumptions or justify their validity | To assess the causal relationship between gut microflora and outcomes, MR analysis was conducted using three methods: inverse variance weighting (IVW), MR-Egger regression, and weighted median (WME). These methods have been extensively described in previous studies. IVW analysis was considered the most reliable method for estimating the causal relationship between exposure and outcome while being sensitive to pleiotropy. MR-Egger regression and WME were further applied as complementary analyses. MR-Egger regression helps detect potential bias in meta-analyses and assesses bias in polymorphism. WME provides consistent effect estimates in MR analyses even though up to half of the information comes from invalid IVs. |
| 8 | **Sensitivity analyses and additional analyses** | Describe any sensitivity analyses or additional analyses performed (e.g. comparison of effect estimates from different approaches, independent replication, bias analytic techniques, validation of instruments, simulations) | To ensure the robustness of the results, a series of sensitivity analyses were performed. Cochran's Q statistics were used to assess heterogeneity among IVs, concluding no heterogeneity when P>0.05. Horizontal polytropic was tested using MR-Egger regression, examining the intercept term to evaluate its impact on the MR analysis results. Leave-one-out analysis was used to determine if any of the SNPs were driving the causal estimates. Finally, the MR Pleiotropy Residual Sum and Outlier (MR-PRESSO) test was employed to identify and correct for outliers in the IVW linear regression, eliminating SNPs associated with heterogeneity. |
| 9 | **Software and pre-registration** |  |  |
|  | a) | Name statistical software and package(s), including version and settings used | To assess potential confounding effects, the selected SNPs were evaluated for associations with other phenotypes using the publicly available PhenoScanner V2 (http://www.phenoscanner.  medschl.cam.ac.uk) |
|  | b) | State whether the study protocol and details were pre-registered (as well as when and where) | No pre-registration involved. |
|  | **RESULTS** |  |  |
| 10 | **Descriptive data** |  |  |
|  | a) | Report the numbers of individuals at each stage of included studies and reasons for exclusion. Consider use of a flow diagram | Summary statistics of the gut microbiota were obtained from a large-scale multi-ethnic GWAS meta-analysis, involving 18340 individuals from 24 cohorts, most of which were of European ancestry. Genus-level taxa were analyzed, with a total of 131 genera showing an average abundance greater than 1%. However, 12 of these genera were unknown, leaving 119 genus-level taxa included in this study. Covariate adjustments were made for gender, age, technical covariates, and genetic principal components using Spearman correlation analysis.  The outcome datasets were extracted from the large publicly available GWAS analysis. Summary data for DM were obtained from a GWAS study of European ancestry, including 208 cases and 213,145 controls. For polymyositis (PM), the study included 119 cases and 213,145 controls. |
|  | b) | Report summary statistics for phenotypic exposure(s), outcome(s), and other relevant variables (e.g. means, SDs, proportions) | **Table 1** Significant MR analysis results in the discovery of Gut Microbiota  **Table 3** Correlation of heterogeneity and pleiotropy tests for polymyositis and dermatomyositis with genetic predictors of Gut Microbiota |
|  | c) | If the data sources include meta-analyses of previous studies, provide the assessments of heterogeneity across these studies | no |
|  | d) | For two-sample MR:  i.  Provide justification of the similarity of the genetic variant-exposure associations between the exposure and outcome samples  ii.  Provide information on the number of individuals who overlap between the exposure and outcome studies | Detailed information on the specific SNPs used in the analysis is provided in the Supplement materials. |
| 11 | **Main results** |  |  |
|  | a) | Report the associations between genetic variant and exposure, and between genetic variant and outcome, preferably on an interpretable scale | As shown in **Table 1** and **Figure2**, we observed a certain genus-level taxa of gut microbiota have causal effects on the risk of PM and DM. Specifically, a higher genetically predicted level of *Alloprevotella* (OR: 2.626, 95% CI: 1.095-6.298, P=0.031) and *Ruminococcaceae UCG003* (OR: 4.219, 95% CI: 1.227-14.511, P=0.022) was associated with an increased risk of PM, while *Dialister* was found to be related to a lower risk of PM (OR:0.273, 95% CI:0.077-0.974, P=0.045). For DM, genus *Anaerotruncus* showed a negative correlation with DM (OR: 0.314, 95%CI: 0.112-0.882, P=0.028), indicating a lower risk of DM, while *Ruminococcaceae UCG002* (OR:2.439, 95% CI:1.173-5.071, P=0.017) and *Sutterella* (OR:3.392, 95% CI:1.302-8.839, P=0.012) were associated with an increased risk of DM. Detailed information on the specific SNPs used in the analysis is provided in the Supplement materials. |
|  | b) | Report MR estimates of the relationship between exposure and outcome, and the measures of uncertainty from the MR analysis, on an interpretable scale, such as odds ratio or relative risk per SD difference | None of the MR-Egger regression intercepts deviated from zero, indicating no evidence of horizontal pleiotropy (all intercepts P > 0.05). Additionally, the leave-one-out analysis demonstrated consistent causal estimations, suggesting that none of the identified causal associations were heavily driven by any single IV. The results of the sensitivity analysis are presented in Table 3 and Fig 4. |
|  | c) | If relevant, consider translating estimates of relative risk into absolute risk for a meaningful time period | None of the MR-Egger regression intercepts deviated from zero, indicating no evidence of horizontal pleiotropy (all intercepts P > 0.05). Additionally, the leave-one-out analysis demonstrated consistent causal estimations, suggesting that none of the identified causal associations were heavily driven by any single IV. The results of the sensitivity analysis are presented in Table 3 and Fig 4. |
|  | d) | Consider plots to visualize results (e.g. forest plot, scatterplot of associations between genetic variants and outcome versus between genetic variants and exposure) | As shown in Table 1 and Fig 3, we observed a certain genus-level taxa of gut microbiota have causal effects on the risk of PM and DM.  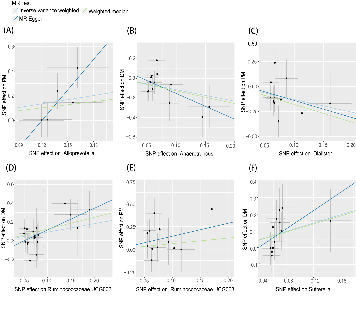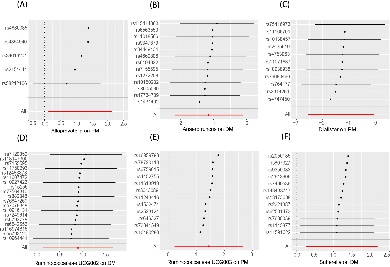 |
| 12 | **Assessment of assumptions** |  |  |
|  | a) | Report the assessment of the validity of the assumptions | To assess the causal relationship between gut microflora and outcomes, MR analysis was conducted using three methods: inverse variance weighting (IVW), MR-Egger regression, and weighted median (WME). These methods have been extensively described in previous studies. IVW analysis was considered the most reliable method for estimating the causal relationship between exposure and outcome while being sensitive to pleiotropy. MR-Egger regression and WME were further applied as complementary analyses. MR-Egger regression helps detect potential bias in meta-analyses and assesses bias in polymorphism. WME provides consistent effect estimates in MR analyses even though up to half of the information comes from invalid IVs. |
|  | b) | Report any additional statistics (e.g., assessments of heterogeneity across genetic variants, such as *I^2^*, Q statistic or E-value) | To ensure the robustness of the results, a series of sensitivity analyses were performed. Cochran's Q statistics were used to assess heterogeneity among IVs, concluding no heterogeneity when P>0.05(26). Horizontal polytropic was tested using MR-Egger regression, examining the intercept term to evaluate its impact on the MR analysis results. Leave-one-out analysis was used to determine if any of the SNPs were driving the causal estimates. Finally, the MR Pleiotropy Residual Sum and Outlier (MR-PRESSO) test was employed to identify and correct for outliers in the IVW linear regression, eliminating SNPs associated with heterogeneity. |
| 13 | **Sensitivity analyses and additional analyses** |  |  |
|  | a) | Report any sensitivity analyses to assess the robustness of the main results to violations of the assumptions | To ensure the robustness of the results, a series of sensitivity analyses were performed. Cochran's Q statistics were used to assess heterogeneity among IVs, concluding no heterogeneity when P>0.05. Horizontal polytropic was tested using MR-Egger regression, examining the intercept term to evaluate its impact on the MR analysis results. Leave-one-out analysis was used to determine if any of the SNPs were driving the causal estimates. Finally, the MR Pleiotropy Residual Sum and Outlier (MR-PRESSO) test was employed to identify and correct for outliers in the IVW linear regression, eliminating SNPs associated with heterogeneity. |
|  | b) | Report results from other sensitivity analyses or additional analyses | None of the MR-Egger regression intercepts deviated from zero, indicating no evidence of horizontal pleiotropy (all intercepts P > 0.05). Additionally, the leave-one-out analysis demonstrated  consistent causal estimations, suggesting that none of the identified causal associations were heavily driven by any single IV. The results of the sensitivity analysis are presented in Table 3 and Fig 4. |
|  | c) | Report any assessment of direction of causal relationship (e.g., bidirectional MR) | Specifically, a higher genetically predicted level of *Alloprevotella* (OR: 2.626, 95% CI: 1.095-6.298, P=0.031) and *Ruminococcaceae UCG003* (OR: 4.219, 95% CI: 1.227-14.511, P=0.022) was associated with an increased risk of PM, while *Dialister* was found to be related to a lower risk of PM (OR:0.273, 95% CI:0.077-0.974, P=0.045). For DM, genus *Anaerotruncus* showed a negative correlation with DM (OR: 0.314, 95%CI: 0.112-0.882, P=0.028), indicating a lower risk of DM, while *Ruminococcaceae UCG002* (OR:2.439, 95% CI:1.173-5.071, P=0.017) and *Sutterella* (OR:3.392, 95% CI:1.302-8.839, P=0.012) were associated with an increased risk of DM. |
|  | d) | When relevant, report and compare with estimates from non-MR analyses | This study employs a two-sample Mendelian randomization (MR) analysis, utilizing summary statistics from Genome Wide Association Studies (GWAS), to explore the potential causal effect of gut microbiota on PM and DM. Single nucleotide polymorphisms (SNPs) significantly associated with the gut microbiota were selected as instrumental variables (IVs), and three assumptions were made to ensure accuracy. First, the genetic variants selected as IVs should be significantly associated with gut microbiota. Second, IVs should not be associated with any confounding factors. Third, IVs should affect the risk of PM and DM only through gut microbiota. A series of sensitivity analyses were then conducted for significant associations. The overall flowchart of the study is presented in **Figure1**. |
|  | e) | Consider additional plots to visualize results (e.g., leave-one-out analyses) | The results of the sensitivity analysis are presented in Table 3 and Fig 4.  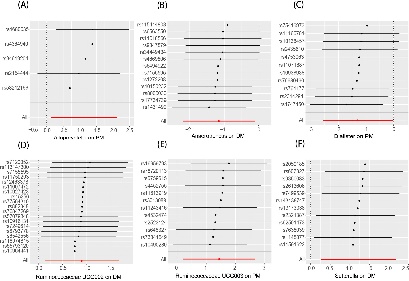 |
|  | **DISCUSSION** |  |  |
| 14 | **Key results** | Summarize key results with reference to study objectives | This study represents the first attempt to investigate the causal relationship between gut microbiota and PM/DM using a two-sample MR analysis. Our findings reveal five different bacterial genera (*Alloprevotella, Ruminococcaceae, Anaerotruncus, Sutterella,* and *Dialister*) that are associated with PM and DM. Notably, the genus *Ruminococcaceae* was found to be causally involved in both conditions, which may allow us to early prevention strategies rather than just treating the disease after it has progressed |
| 15 | **Limitations** | Discuss limitations of the study, taking into account the validity of the IV assumptions, other sources of potential bias, and imprecision. Discuss both direction and magnitude of any potential bias and any efforts to address them | Indeed, certain limitations in our study should be acknowledged. Firstly, our analysis of the intestinal flora was conducted at the genus level, and a more detailed analysis at the species or strain level could provide deeper insights and improve the accuracy of the findings. By examining specific bacterial species or strains, we could potentially identify more precise associations with PM and DM. Additionally, it is important to consider the age differences in the occurrence of PM and DM. DM shows a bimodal pattern of incidence, with peaks in childhood and later between the ages of 50 and 70 years. On the other hand, PM is rare in childhood and typically occurs after the second decade of life. Furthermore, both conditions are more common in females. Gender differences are known to exist in autoimmune disorders, but our study did not explore the gender-specific effects of the gut microbiota on PM and DM. Future research should aim to investigate these potential differences and explore the specific associations between gut microbiota, gender, and PM/DM. Another aspect to be considered is the threshold used for statistical significance. In our study, we employed a P-value threshold of P<1×10^-5^, which may have resulted in some significant associations being missed. Future studies aiming to establish a causal relationship between gut bacteria and PM/DM should consider using a more stringent threshold to ensure robust findings. |
| 16 | **Interpretation** |  |  |
|  | a) | Meaning: Give a cautious overall interpretation of results in the context of their limitations and in comparison with other studies | This study represents the first attempt to investigate the causal relationship between gut microbiota and PM/DM using a two-sample MR analysis. |
|  | b) | Mechanism: Discuss underlying biological mechanisms that could drive a potential causal relationship between the investigated exposure and the outcome, and whether the gene-environment equivalence assumption is reasonable. Use causal language carefully, clarifying that IV estimates may provide causal effects only under certain assumptions | Several studies have indicated that butyrate-producing bacteria promote the development of regulatory T cells (Tregs)in the gut-associated lymphatic system, which helps restore immune homeostasis and reduce the risk of autoimmune disease pathogenesis. Additionally, butyrate production by *Ruminococcaceae* is known to enhance gut health, support epithelial cell function and morphology, and regulate the balance of intestinal flora.However, it is important to note that butyrate production by *Ruminococcaceae* inhibits histone deacetylase (HDAC), which can affect the accumulation of immune-related molecules and CD8+ T cells. The increase in CD8+ T cells has been associated with muscle fiber destruction observed in PM, suggesting a potential mechanism linking *Ruminococcaceae* and an increased risk of PM. Furthermore, evidence from observational studies, MR analyses, and clinical trials suggests that the effects of *Ruminococcaceae* may vary depending on the specific species and strain. Genomic analysis has revealed that certain *Ruminococcaceae* strains associated with autoimmune diseases are distinct from those found in healthy individuals. However, further investigation at a specialized genomic level is necessary to fully understand this new finding. |
|  | c) | Clinical relevance: Discuss whether the results have clinical or public policy relevance, and to what extent they inform effect sizes of possible interventions | Through our research, we discovered that the relative abundance of *Ruminococcaceae* may be related to disease activity in PM as well as DM. Although *Ruminococcaceae* are commonly found in low levels in the feces of healthy populations, even patients with mild disease activity showed higher-than-normal levels of *Ruminococcaceae*. We observed that patients with high disease activity had even higher levels of *Ruminococcaceae*, leading us to hypothesize that increased activity of this bacterium could raise the risk of developing PM/DM. Considering this association, monitoring the abundance of *Rumenococcaceae* flora at an early stage can provide targeted recommendations for preventive and therapeutic approaches. |
| 17 | **Generalizability** | Discuss the generalizability of the study results (a) to other populations, (b) across other exposure periods/timings, and (c) across other levels of exposure | The strength of this study lies in the early monitoring of risk factors and the availability of effective treatment strategies that can minimize the burden of healthcare costs and disease suffering. Our study utilized MR to use genetic letters as IVs, thereby predicting the relationship between gut flora and PM/DM at the genetic level. The strengths of our study compared with previous studies are threefold: first, it is the first attempt of its kind, giving us more possibilities to study this type of disease; second, it lies in our rigorous selection of IVs and thorough sensitivity analyses to ensure the validity of our causal estimates. During the IV selection process, we employed stringent quality control measures, including the use of independent GW AS SNPs and the assessment of horizontal pleiotropy; finally, the large sample size of this study and the European origin of the participants further enhanced the reliability of our results. These advantages help to deepen our understanding of the relationship between gut flora and PM/DM and facilitate the development of therapeutic strategies for PM/DM. |
|  | **OTHER INFORMATION** |  |  |
| 18 | **Funding** | Describe sources of funding and the role of funders in the present study and, if applicable, sources of funding for the databases and original study or studies on which the present study is based | This project was supported by grants from the National Natural Science Foundation of China (NO.82001740) and the Natural Science Foundation of Shanxi Province (NO.202203021221269). |
| 19 | **Data and data sharing** | Provide the data used to perform all analyses or report where and how the data can be accessed, and reference these sources in the article. Provide the statistical code needed to reproduce the results in the article, or report whether the code is publicly accessible and if so, where | GWAS summary statistics about Dermatomyositis and Polymyositis are available in the IEU OpenGWAS project(https://gwas.mrcieu.ac.uk/datasets/finn-b-M13_DERMATOPOLY/and https://gwas.mrcieu.ac.uk/datasets/finn-b-M13_POLYMYO/). And the data of gut microbiota is publicly available and its original studies are cited from (<https://www.nature.com/articles/s41588-020-00763-1>) |
| 20 | **Conflicts of Interest** | All authors should declare all potential conflicts of interest | The authors declare no competing interests. |

This checklist is copyrighted by the Equator Network under the Creative Commons Attribution 3.0 Unported (CC BY 3.0) license.

1. Skrivankova VW, Richmond RC, Woolf BAR, Yarmolinsky J, Davies NM, Swanson SA, et al. Strengthening the Reporting of Observational Studies in Epidemiology using Mendelian Randomization (STROBE-MR) Statement. JAMA. 2021;under review.

2. Skrivankova VW, Richmond RC, Woolf BAR, Davies NM, Swanson SA, VanderWeele TJ, et al. Strengthening the Reporting of Observational Studies in Epidemiology using Mendelian Randomisation (STROBE-MR): Explanation and Elaboration. BMJ. 2021;375:n2233.
